# Comparative Analysis of *C9orf72* and Sporadic Disease in a Large Multicenter ALS Population: The Effect of Male Sex on Survival of *C9orf72* Positive Patients

**DOI:** 10.3389/fnins.2019.00485

**Published:** 2019-05-17

**Authors:** Francesca Trojsi, Mattia Siciliano, Cinzia Femiano, Gabriella Santangelo, Christian Lunetta, Andrea Calvo, Cristina Moglia, Kalliopi Marinou, Nicola Ticozzi, Christian Ferro, Carlo Scialò, Gianni Sorarù, Amelia Conte, Yuri M. Falzone, Rosanna Tortelli, Massimo Russo, Valeria Ada Sansone, Adriano Chiò, Gabriele Mora, Vincenzo Silani, Paolo Volanti, Claudia Caponnetto, Giorgia Querin, Mario Sabatelli, Nilo Riva, Giancarlo Logroscino, Sonia Messina, Antonio Fasano, Maria Rosaria Monsurrò, Gioacchino Tedeschi, Jessica Mandrioli

**Affiliations:** ^1^Department of Advanced Medical and Surgical Sciences, MRI Research Center SUN-FISM, University of Campania “Luigi Vanvitelli”, Naples, Italy; ^2^Department of Psychology, Università degli Studi della Campania “L. Vanvitelli”, Naples, Italy; ^3^NEuroMuscular Omnicentre (NEMO), Serena Onlus Foundation, Milan, Italy; ^4^NEMO Sud Clinical Center for Neuromuscular Diseases, Aurora Onlus Foundation, Messina, Italy; ^5^ALS Center, “Rita Levi Montalcini” Department of Neuroscience, University of Torino, Turin, Italy; ^6^Department of Neurorehabilitation–ALS Center, IRCCS Scientific Clinical Institute Maugeri, Milan, Italy; ^7^Department of Neurology and Laboratory of Neuroscience, IRCCS Istituto Auxologico Italiano, Milan, Italy; ^8^Department of Pathophysiology and Transplantation, “Dino Ferrari” Center, University of Milan, Milan, Italy; ^9^Neurorehabilitation Unit/ALS Center, Scientific Clinical Institutes (ICS) Maugeri, IRCCS, Messina, Italy; ^10^Department of Neurosciences, Rehabilitation, Ophthalmology, Genetics, Maternal, and Child Health (DINOGMI), University of Genova, IRCCS AOU San Martino-IST, Genova, Italy; ^11^Department of Neurosciences, Neuromuscular Center, University of Padova, Padua, Italy; ^12^NEuroMuscular Omnicentre (NEMO), Serena Onlus Foundation–Pol. A. Gemelli Foundation, Rome, Italy; ^13^Department of Neurology, Institute of Experimental Neurology (INSPE), Division of Neuroscience, San Raffaele Scientific Institute, Milan, Italy; ^14^Department of Clinical Research in Neurology, University of Bari “A. Moro”, at Pia Fondazione “Card. G. Panico”, Lecce, Italy; ^15^Department of Clinical and Experimental Medicine, University of Messina, Messina, Italy; ^16^Department of Biomedical Sciences for Health, University of Milan, Milan, Italy; ^17^Department of Geriatrics, Neurosciences and Orthopedics, Institute of Neurology, Catholic University of Sacred Heart, Rome, Italy; ^18^Department of Neuroscience, S. Agostino-Estense Hospital and University of Modena and Reggio Emilia, Modena, Italy

**Keywords:** amyotrophic lateral sclerosis, *C9orf72* expansion, gender, comorbidity, survival

## Abstract

We investigated whether the *C9orf72* repeat expansion is associated with specific clinical features, comorbidities, and prognosis in patients with amyotrophic lateral sclerosis (ALS). A cohort of 1417 ALS patients, diagnosed between January 1, 2009 and December 31, 2013 by 13 Italian ALS Referral Centers, was screened for the *C9orf72* repeat expansion, and the analyses were performed comparing patients carrying this expansion (ALS-C9Pos) to those negative for this and other explored ALS-related mutations (ALS without genetic mutations, ALSwoGM). Compared to the ALSwoGM group, ALS-C9Pos patients (*n* = 84) were younger at disease onset, at the first clinical observation and at diagnosis (*p* < 0.001). After correcting for these differences, we found that ALS-C9Pos patients had higher odds of bulbar onset, diagnosis of frontotemporal dementia (FTD) and family history of ALS, FTD, and Alzheimer's disease and had lower odds of spinal onset, non-invasive ventilation, hypertension and psychiatric diseases than ALSwoGM patients. Among these variables, those related to shorter survival time were: bulbar onset, presence of FTD, hypertension, psychiatric disease, and family history of ALS (*p* < 0.05). Cox proportional hazards regression multivariate analysis suggested that carrying the *C9orf72* repeat expansion was an independent factor negatively impacting on survival time in men (HR 1.58, 95% CI 1.07–2.33, *p* = 0.021), but not in women (*p* > 0.05) as well as in the whole sample (*p* > 0.05). When compared to ALSwoGM, ALS-C9Pos showed an earlier disease onset, no significant diagnostic delay and a higher odds of bulbar onset, FTD and family history of ALS and dementia. Moreover, male sex drove the negative effect of expanded variant on survival, confirming the hypothesis that sex is likely to be a crucial factor in the biology of *C9orf72*-related disease.

## Introduction

The pathological expansion of a hexanucleotide repeat in the *C9orf72* gene is the most common genetic mutation identified in patients with amyotrophic lateral sclerosis (ALS), reported in 40–50% of patients with familial ALS and 5–10% of patients with sporadic ALS (DeJesus-Hernandez et al., 2011; Renton et al., 2011; Majounie et al., 2012). Patients carrying the expansion (ALS-C9Pos) have been described as phenotypically different from non-mutated patients in that most cohorts of ALS-C9Pos patients exhibited, at variable extent, higher prevalence of bulbar onset, earlier age at onset and reduced survival with higher incidence of comorbid frontotemporal dementia (FTD) and/or family history of dementia or ALS (Byrne et al., 2012, 2013; Chiò et al., 2012; Cooper-Knock et al., 2012; Majounie et al., 2012; Sabatelli et al., 2012; Irwin et al., 2013; Umoh et al., 2016; Hardiman et al., 2017). Although the *C9orf72* repeat expansion has been revealed a relevant negative prognostic factor in survival analyses (Byrne et al., 2012, 2013; Chiò et al., 2012; Sabatelli et al., 2012; Irwin et al., 2013; Umoh et al., 2016), the potential associations between this variant and demographic and clinical features have not still completely elucidated. Among the most robust evidence on prognostic char (Rooney et al., 2017) a previously unrecognized interaction between the *C9orf72* repeat expansion and sex (Rooney et al., 2017). Nevertheless, the same authors suggested to further evaluate the role of other clinical variables, such as the presence of cognitive changes, in the abovementioned interaction effect. Additionally, Miltenberger-Miltenyi et al. (2018) found that the *C9orf72* expansion was associated with FTD, shorter survival and faster % Forced Vital Capacity (FVC) decline in ALS, but not with a faster rate of functional decay.

In this large multicenter cohort we aimed at: (i) examining the potential associations between *C9orf72* repeat expansion and phenotype, site of onset, family history, therapy, and others comorbidities; (ii) exploring if ALS patients carrying *C9orf72* repeat expansion differed from patients without genetic mutation in the survival profile, both in the whole sample and stratified by sex. We expected to identify potential novel associations between *C9orf72* repeat expansion and a number of demographic and clinical features, focusing on the effect of sex on the survival profile.

## Materials and Methods

### Patient Data Collection

**Figure 1:**
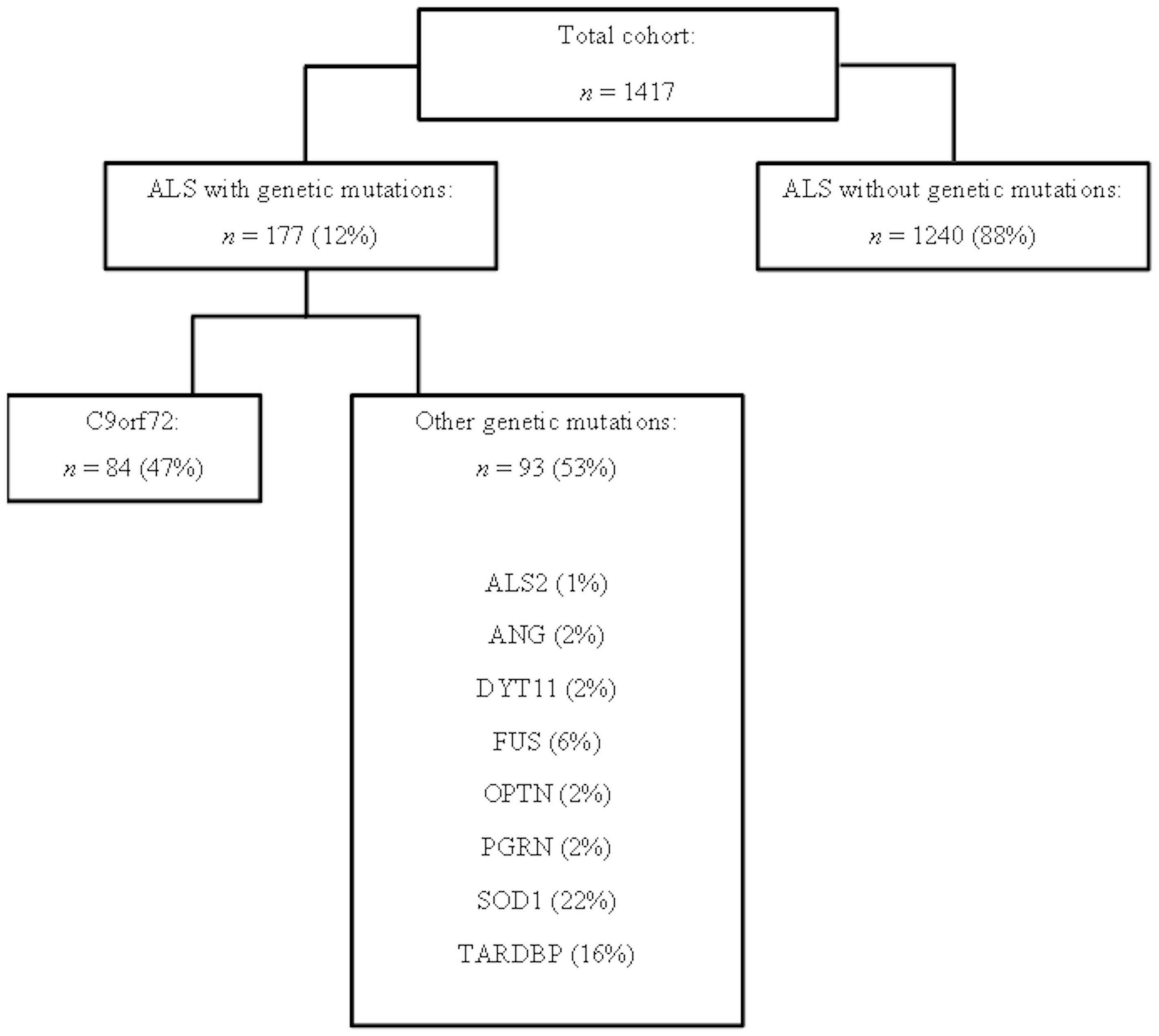
Flow chart representing the studied cohort from 13 ALS Italian referral centers, in which genetic data were available.

This study has been designed and performed in 13 ALS Italian referral centers, located in 10 Italian Regions: ALS Centers of Turin, Padua, Genoa, Naples, Modena, Lecce, Rome, NEMO Clinical Centers in Milan, and Messina, ALS Centers of ICS Maugeri in Milan and Mistretta, ALS Centers at San Raffaele Institute, and IRCCS Istituto Auxologico Italiano in Milan (Calvo et al., 2017; Trojsi et al., 2017b; Mandrioli et al., 2018a). We included 1417 patients, diagnosed with definite or clinical and laboratory-supported probable ALS, according to the Revised El Escorial Criteria (Brooks et al., 2000), from January 1st, 2009 to December 31st, 2013, in whom genetic data were available (Figure 1).

Data have been recorded into an electronic database available to all involved centers. According to previously used selection methods (Umoh et al., 2016), all patients followed at the involved ALS referral centers were consecutively asked to donate DNA for research purposes, and the only criteria for inclusion were the diagnosis of ALS and the consent to donate blood for genetic screening. Caring neurologists collected a detailed phenotypic profile for each ALS patient, including the following information: among demographic data, sex, age at onset, at clinical observation and at diagnosis; among clinical data, site, and time of onset, clinical phenotype [classic, bulbar, predominant upper motor neuron (UMN-p), flail arm, flail leg, and respiratory ALS] (Chiò et al., 2011), presence of concomitant dementia and family history of ALS, FTD or other neurodegenerative diseases (i.e., Parkinson's and Alzheimer's disease), metabolic (i.e., diabetes), oncologic, cardiovascular (i.e., hypertension, atrial fibrillation, and heart failure), auto-immune, hematological, gastroenteric, and psychiatric diseases. The genetic analysis included screening for *SOD1, FUS, TARDBP*, and *C9orf72* status (normal or expanded), four genes accounting for up to 70% of all cases of familial ALS (Hardiman et al., 2017). When mutations of these genes or *C9orf72* expansion were not revealed and in presence of family history of ALS and/or FTD, mutations of *ALS2, ANG, DYT11, OPTN*, and *PGRN* were also explored. *C9orf72* status was determined by repeat primed PCR as described previously (with individual laboratory-based validation and quality control by Southern blot analyses) (DeJesus-Hernandez et al., 2011; Renton et al., 2011; Byrne et al., 2012, 2013; Chiò et al., 2012; Cooper-Knock et al., 2012; Majounie et al., 2012; Sabatelli et al., 2012; Irwin et al., 2013; Umoh et al., 2016). Among outcome information, dates of percutaneous endoscopic gastrostomy (PEG), non-invasive ventilation (NIV) and tracheotomy/death were also collected.

This study was approved by the Ethical Committees of the participating ALS centers and conducted according to the principles expressed in the Declaration of Helsinki. Patient or family written consent was obtained from each participant.

### Statistical Analysis

Descriptive statistics are reported as count and percentage for categorical variables (i.e., sex) or mean and standard deviation for continuous variables (i.e., age at onset, age at clinical observation, age at diagnosis and diagnostic delay).

The comparisons between ALS-C9Pos and ALS patients without genetic mutations (ALSwoGM) were performed using one-way analysis of variance (ANOVA), and Pearson chi-square test (χ^2^), when appropriate.

Logistic regression analyses (hierarchical method) were used for measuring the association between the presence of *C9orf72* expansion and clinical phenotype (i.e., classic, bulbar, flail arm, flail leg, and UMN-p), site of onset (i.e., spinal and bulbar), therapeutic interventions (i.e., NIV, PEG, tracheostomy, and riluzole), other diseases (i.e., FTD, diabetes, hypertension, heart disease, cancer, autoimmune, hematological, gastroenteric, and psychiatric), and family history (i.e., ALS, FTD, and Parkinson's and Alzheimer's disease). All logistic regression models were adjusted for baseline demographic and clinical differences between ALS-C9Pos and ALSwoGM, and the results were presented as adjusted odds ratio (OR). Kaplan-Meier univariate analysis was used to determine the effect of *C9orf72* repeat expansion on the survival time for the whole sample as well as for the men and the women. Moreover, the univariate effect on survival time of the variables associated to the presence of *C9orf72* repeat expansion was explored by Kaplan-Meier analysis for categorical variables (using the log-rank test) and by Cox proportional hazards analysis for continuous variables (using hazard ratios or HR, and 95% confidence interval or 95% CI).

Finally, Cox proportional hazards regression multivariate analysis (Forward Conditional method) was performed entering the variables associated with the survival time in univariate analyses in order to explore the effect of the *C9orf72* repeat expansion on survival time in a multivariate model. These analyses were performed in the whole sample and, then, stratifying by sex. Survival time was defined as time from symptom onset to time of death/tracheotomy. Patients who were alive at time of analysis were censored.

Statistical analyses were performed using IBM Statistical Package for Social Science (SPSS) version 20, with *p*-value < 0.05.

## Results

The *C9orf72* repeat expansion was identified in 84 cases (Figure 1). ALSwoGM were 1240, excluding 93 ALS patients carrying other genetic mutations (Figure 1).

Comparing ALS-C9Pos to ALSwoGM patients, there were significant differences in age at onset, age at clinical observation, and age at diagnosis (Table 1).

**Table 1:** Descriptive statistics of amyotrophic lateral sclerosis patients with pathogenic *C9orf72* expansion (ALS-C9Pos) and without genetic mutations (ALSwoGM).

	**ALS-C9Pos (*n* = 84)**	**ALSwoGM (*n* = 1240)**	**F/χ^2^**	***p***
Sex, *n* (%)				
Male	47 (54%)	681 (55%)	0.03	0.854
Female	37 (46%)	559 (45%)		
Mean age at onset, years (SD)	58.49 (9.55)	63.41 (11.6)	14.44	**<0.001**
Mean age at clinical observation, years (SD)	64.36 (9.78)	69.82 (11.47)	18.10	**<0.001**
Mean age at diagnosis, years (SD)	59.47 (9.55)	64.69 (11.44)	16.69	**<0.001**
Diagnostic delay, months (SD)	11.73 (8.58)	15.35 (21.85)	2.28	0.131

*ALS-C9Pos, amyotrophic lateral sclerosis with pathogenic C9orf72 expansion; ALSwoGM, amyotrophic lateral sclerosis without genetic mutations; significant differences are signed in **bold**; SD, standard deviation*.

After having adjusted for demographic and clinical differences, logistic regression analyses showed that pathogenic *C9orf72* expansion was associated with higher OR of bulbar onset, FTD diagnosis, and family history of ALS, FTD, and Alzheimer's disease. In addition, the *C9orf72* expansion was associated with lower OR of spinal onset, NIV, hypertension, and psychiatric diseases (Table 2). Noteworthy, no ALS patients with pathogenic *C9orf72* expansion had respiratory phenotype or respiratory onset.

**Table 2:** Relationship between pathogenic *C9orf72* expansion and phenotype, site onset, family history, therapy, and others comorbidities in patients with amyotrophic lateral sclerosis.

	**ALS-C9Pos**	**ALSwoGM**	***p***	**OR**	**95%CI**
	***n (%)***	***n (%)***			
**Classic phenotype**					
Yes	53 (63%)	633 (51%)	0.132	1.42	[0.89, 2.26]
No	31 (37%)	607 (49%)			
**Bulbar phenotype**					
Yes	16 (19%)	224 (18%)	0.434	1.25	[0.71, 2.24]
No	68 (81%)	1016 (82%)			
**Flail arm phenotype**					
Yes	1 (1%)	79 (6%)	0.102	0.19	[0.02, 1.39]
No	83 (99%)	1161 (94%)			
**Flail leg phenotype**					
Yes	1 (1%)	76 (6%)	0.113	0.20	[0.02, 1.46]
No	83 (99%)	1164 (94%)			
**Umn phenotype**					
Yes	5 (6%)	108 (9%)	0.375	0.65	[0.25, 1.66]
No	79 (94%)	1132 (91%)			
**Spinal onset**					
Yes	51 (61%)	881 (71%)	**0.009**	0.53	[0.33, 0.85]
No	33 (39%)	358 (29%)			
**Bulbar onset**					
Yes	32 (38%)	346 (28%)	**0.012**	1.83	[1.14, 2.93]
No	52 (62%)	893 (72%)			
**NIV**					
Yes	26 (31%)	580 (47%)	**0.015**	0.55	[0.33, 0.89]
No	58 (69%)	660 (53%)			
**PEG**					
Yes	31 (37%)	415 (34%)	0.377	1.23	[0.77, 1.98]
No	53 (63%)	825 (66%)			
**Tracheostomy**					
Yes	17 (20%)	203 (16%)	0.386	1.28	[0.73, 2.24]
No	67 (80%)	1037 (84%)			
**Riluzole**					
Yes	74 (88%)	1043 (84%)	0.591	1.20	[0.61, 2.4]
No	10 (12%)	197 (16%)			
**FTD**					
Yes	22 (26%)	93 (7%)	**<0.001**	5.41	[3.10, 9.45]
No	62 (74%)	1147 (93%)			
**Diabetes**					
Yes	3 (4%)	116 (9%)	0.143	0.41	[0.13, 1.34]
No	81 (96%)	1124 (91%)			
**Hypertension**					
Yes	19 (23%)	561 (45%)	**0.004**	0.45	[0.26, 0.78]
No	65 (77%)	679 (55%)			
**Heart Diseases**					
Yes	5 (6%)	203 (16%)	0.080	0.43	[0.17, 1.10]
No	79 (94%)	1037 (84%)			
**Cancer**					
Yes	4 (5%)	141 (11%)	0.119	0.44	[0.15, 1.23]
No	80 (95%)	1099 (89%)			
**Autoimmune diseases**					
Yes	6 (7%)	90 (7%)	0.979	0.99	[0.41, 2.35]
No	78 (93%)	1150 (93%)			
**Hematological diseases**					
Yes	1 (1%)	48 (4%)	0.332	0.37	[0.05, 2.74]
No	83 (99%)	1192 (96%)			
**Gastroenteric diseases**					
Yes	7 (8%)	197 (16%)	0.070	0.48	[0.21, 1.06]
No	77 (92%)	1043 (84%)			
**Psychiatric diseases**					
Yes	4 (5%)	143 (12%)	**0.043**	0.35	[0.12, 0.96]
No	80 (95%)	1097 (88%)			
**Family history of ALS**					
Yes	38 (45%)	51 (4%)	**<0.001**	17.26	[10.25, 29.05]
No	46 (55%)	1189 (96%)			
**Family history of PD**					
Yes	3 (4%)	49 (4%)	0.640	0.75	[0.23, 2.49]
No	81 (96%)	1191 (96%)			
**Family history of FTD**					
Yes	15 (18%)	14 (1%)	**<0.001**	16.23	[7.39, 35.62]
No	69 (82%)	1226 (99%)			
**Family history of AD**					
Yes	18 (21%)	100 (8%)	**0.001**	2.70	[1.52, 4.79]
No	66 (79%)	1140 (92%)			

*ALS, amyotrophic lateral sclerosis; ALS-C9Pos, amyotrophic lateral sclerosis with pathogenic C9orf72 expansion; ALSwoGM, amyotrophic lateral sclerosis without genetic mutations; adjOR, adjusted odds ratio; AD, Alzheimer's disease; FTD, frontotemporal dementia; NIV, non-invasive ventilation; PD, Parkinson's disease; PEG, percutaneous endoscopic gastrostomy; significant associations are signed in **bold***.

**Figure 2:**
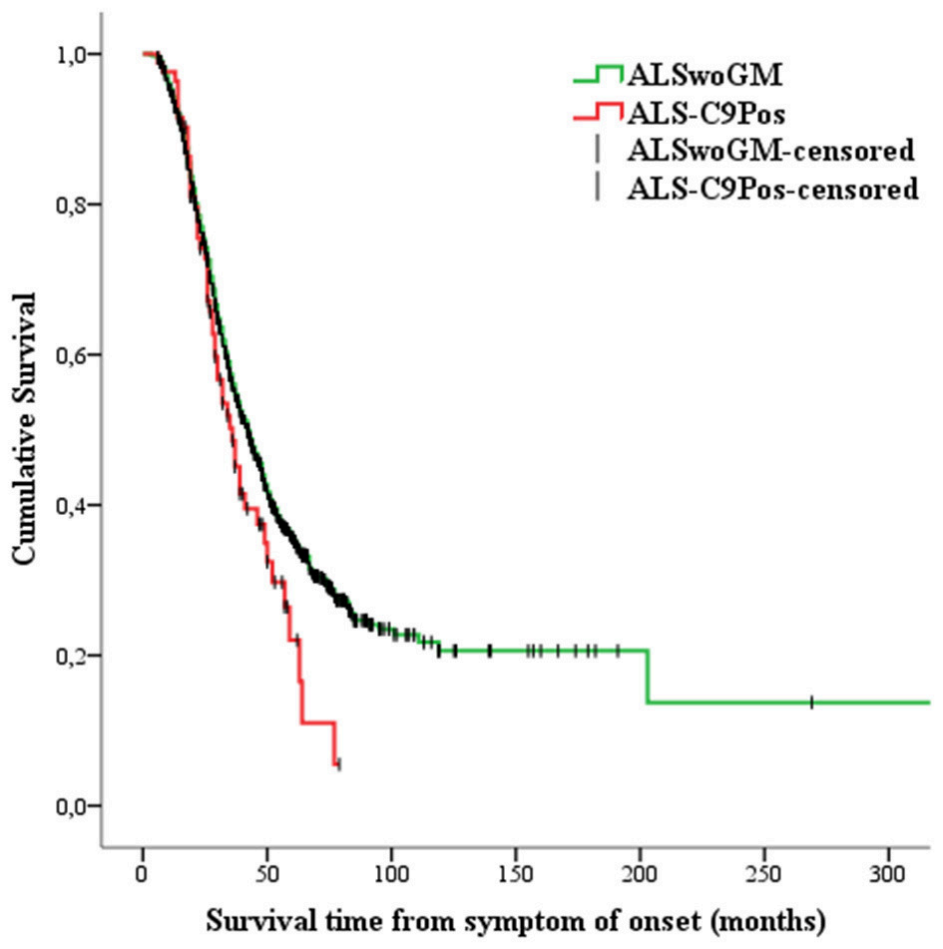
Kaplan-Meier plots of survival probabilities: the patients carrying the pathogenic *C9orf72* repeat expansion (or ALS-C9Pos, red line) display shorter survival time [median survival of 36 months (95% CI 30–43)] than the patients without genetic mutations (ALSwoGM, green line) [median survival of 42 months (95% CI 39–45)]. Log-rank χ^2^ = 3.88, *p* = .049; +: censored cases.

**Figure 3:**
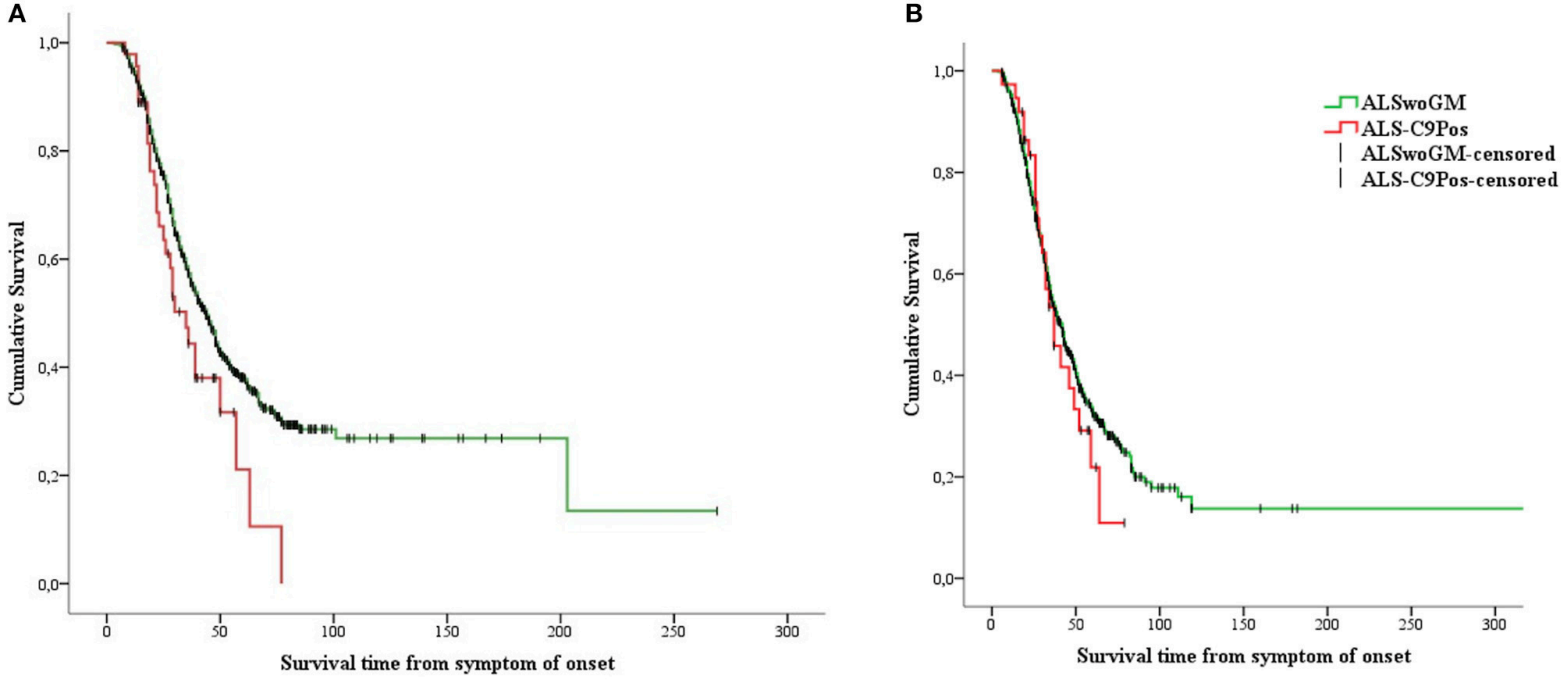
Kaplan-Meier plots of survival probabilities, stratifying the overall sample by sex: shorter survival time is displayed in ALS-C9Pos patients (red line) compared to ALSwoGM patients (green line) only for males. **(A)** (male): Log-rank χ^2^ = 4.33, *p* = 0.037; median survival was 35 months (95% CI 26–44) for ALS-C9Pos (*n* = 47), and 44 months (95% CI 40–48) for ALSwoGM (*n* = 681). **(B)** (female): Log-rank χ^2^ = 0.43, *p* = 0.510; median survival was 37 months (95% CI 26–47) for ALS-C9Pos (*n* = 37), and 42 months (95% CI 37–46) for ALSwoGM (*n* = 559). +: censored cases.

Kaplan-Meier analysis showed that ALS-C9Pos patients had shorter survival time than ALSwoGM patients (Figure 2). When the overall sample was stratified according to sex, this pattern of results was confirmed only for men (Figure 3). However, a preliminary Kaplan-Meier analysis did not show significant differences in the survival time comparing men to women for the overall sample, regardless of the stratification due to the presence of *C9orf72* repeat expansion (Supplemental Figure 1). Among the variables associated with the presence of *C9orf72* expansion, those related to shorter survival time on univariate analyses included the following: (1) higher age at onset (HR 1.04, 95% CI 1.03–1.04, *p* < 0.0001); (2) higher age at clinical observation (HR 1.03, 95% CI 1.02–1.04, *p* < 0.0001); (3) higher age at diagnosis (HR 1.03, 95% CI 1.02–1.04, *p* < 0.0001); (4) site of onset, with worse outcome for bulbar onset (log-rank test, *p* < 0.0001); (5) the presence of FTD (log-rank test, *p* < 0.0001); (6) hypertension (log-rank test, *p* < 0.0001); (7) psychiatric diseases (log-rank test, *p* = 0.047); (8) family history of ALS (log-rank test, *p* = 0.015).

**Figure 4:**
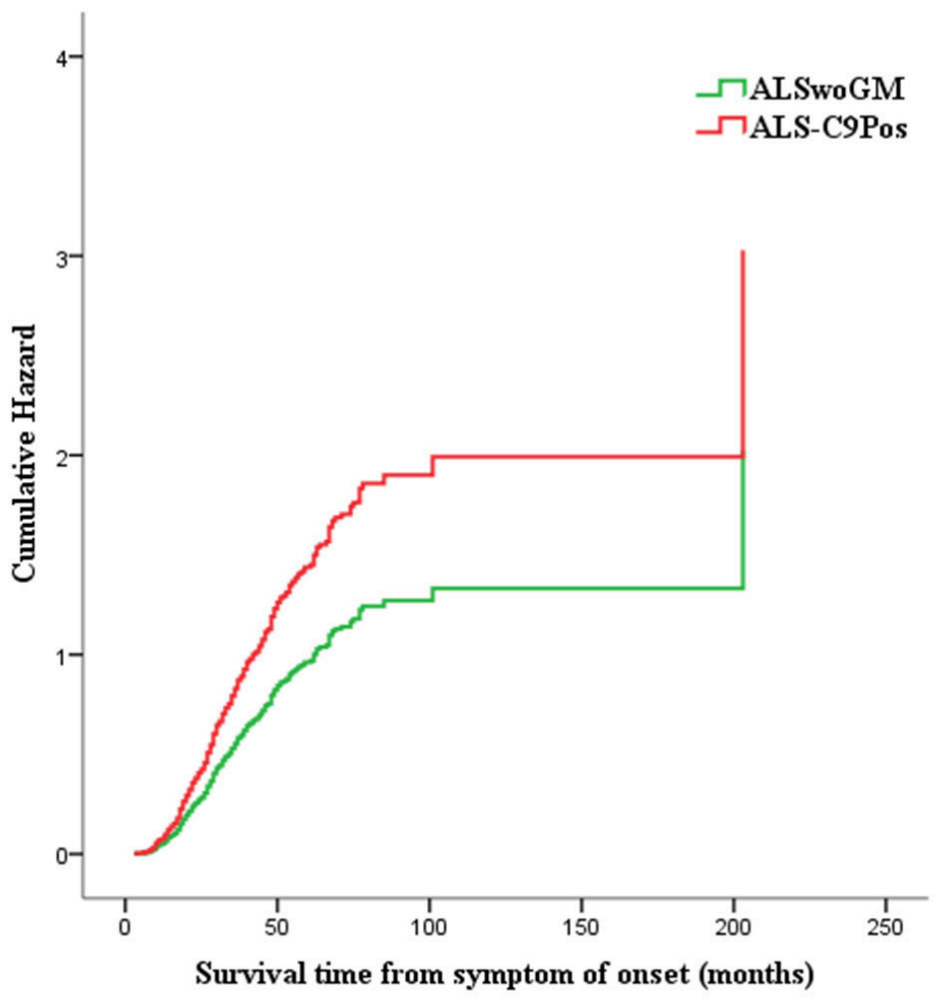
Hazard plot representing the risk of death/tracheotomy for males carrying the pathogenic *C9orf72* repeat expansion (or ALS-C9Pos, red line) compared to males without genetic mutations (or ALSwoGM, green line): shorter survival time is associated with the presence of *C9orf72* repeat expansion (HR 1.58, 95% CI 1.07–2.33, *p* = 0.021).

Cox proportional hazards regression multivariate analysis showed that shorter survival time was associated with the presence of *C9orf72* expansion in men (HR 1.58, 95% CI 1.07–2.33, *p* = 0.021), but not in women (*p* > 0.05) as well as in the whole sample (*p* > 0.05) (Table 3; Figure 4; see also the Supplemental Table 1 for variables that were not included in the equation for each step of the Cox proportional hazards regression multivariate analysis).

**Table 3:** Cox proportional hazards regression multivariate analysis (Forward Conditional method) performed both in the whole sample and stratified by sex.

**Variable**	**B (SE)**	***p*-Value**	**HR**	**95% CI**
**Whole sample**				
*Step 1*				
Age at onset	0.04 (0.00)	< 0.001	1.04	1.03–1.05
*Step 2*				
Age at onset	0.04 (.00)	< 0.001	1.04	1.03–1.05
Diagnostic delay	−0.05 (0.00)	< 0.001	0.95	0.94–0.96
*Step 3*				
Age at onset	0.04 (0.00)	< 0.001	1.04	1.03–1.05
Diagnostic delay	−0.05 (0.00)	< 0.001	0.95	0.94–0.96
FTD	0.46 (0.12)	< 0.001	1.58	1.25–1.99
*Step 4*				
Age at onset	0.03 (0.00)	< 0.001	1.03	1.03–1.04
Diagnostic delay	−0.05 (0.00)	< 0.001	0.95	0.94–0.96
Site of onset (Bulbar = 0; Spinal = 1)	−0.25 (0.08)	0.001	0.77	0.66–0.90
FTD	0.42 (0.12)	< 0.001	1.53	1.21–1.94
*Step 5^a^*				
Age at onset	0.04 (0.00)	< 0.001	1.04	1.03–1.05
Diagnostic delay	−0.05 (0.00)	< 0.001	0.95	0.94–0.96
Site of onset (Bulbar = 0; Spinal = 1)	−0.25 (0.08)	0.002	0.78	0.66–0.91
FTD	0.38 (0.12)	0.002	1.47	1.16–1.86
Family history of ALS	0.31 (0.14)	0.037	1.35	1.01–1.79
**Males**				
*Step 1*				
Diagnostic delay	−0.05 (0.00)	< 0.001	0.95	0.94–0.96
*Step 2*				
Diagnostic delay	−0.06 (0.00)	< 0.001	0.95	0.93–0.96
Age at clinical observation	0.04 (0.00)	< 0.001	1.04	1.03–1.05
*Step 3*				
Diagnostic delay	−0.06 (0.00)	< 0.001	0.95	0.93–0.96
Age at clinical observation	0.04 (0.00)	< 0.001	1.03	1.02–1.05
Site of onset (Bulbar = 0; Spinal = 1)	−0.32 (0.12)	0.005	0.72	0.58–0.91
*Step 4^b^*				
Diagnostic delay	−0.06 (0.00)	< 0.001	0.95	0.93–0.96
Age at clinical observation	0.04 (0.00)	< 0.001	1.04	1.03–1.05
Site of onset (Bulbar = 0; Spinal = 1)	−0.31 (0.12)	0.008	0.73	0.58–0.92
*C9orf72* expansion status (No = 0; SI = 1)	0.46 (0.20)	0.021	1.58	1.07–2.33
**Females**				
*Step 1*				
Age at onset	0.04 (0.00)	< 0.001	1.04	1.03–1.05
*Step 2*				
Age at onset	0.04 (0.00)	< 0.001	1.04	1.03–1.05
Diagnostic delay	−0.05 (0.00)	< 0.001	0.95	0.94–0.96
*Step 3^c^*				
Age at onset	0.04 (0.00)	< 0.001	1.04	1.03–1.05
Diagnostic delay	−05 (0.00)	< 0.001	0.95	0.94–0.96
FTD	0.65 (0.18)	< 0.001	1.91	1.33–2.73

*SE, Standard Error; HR, Hazard Ratio; CI, Confidence Interval; FTD, frontotemporal dementia; ^a^Variables not in equation at last step: age at diagnosis, age at clinical observation, hypertension, psychiatric diseases, and C9orf72 expansion status; ^b^Variables not in equation at last step: age at diagnosis, age at onset, FTD, hypertension, psychiatric diseases, and family history of ALS; ^c^Variables not in equation at last step: age at diagnosis, age at clinical observation, site of onset, hypertension, psychiatric diseases, family history of ALS, and C9orf72 expansion status*.

## Discussion

Patients with ALS carrying the *C9orf72* repeat expansion have been shown to exhibit remarkable clinical and pathological features suggesting that this hexanucleotide expansion identifies a distinct population of patients, with significant implications on therapeutic interventions design and screening for inclusion in clinical trials. In our multicenter analysis, performed on data from 1417 patients, we compared demographic and clinical features of a population of ALS-C9Pos patients to those of a cohort of ALSwoGM patients to determine whether these two groups were phenotypically distinct. Our results partly confirmed previous evidence in that ALS-C9Pos patients were younger at onset, at first clinical observation and at diagnosis and exhibited higher odds of bulbar onset, FTD diagnosis, and family history of ALS, FTD, and Alzheimer's disease. Remarkably, we revealed that ALS-C9Pos patients, especially males, had shorter survival than ALSwoGM patients, thereby enhancing the emerging hypothesis that sex may represent a crucial variable in the pathobiology of *C9orf72*-mediated disease.

Our findings derived from between-groups comparisons mirrored previous results regarding lower age at disease onset (Byrne et al., 2012; Cooper-Knock et al., 2012; Irwin et al., 2013) and diagnosis (Byrne et al., 2012) in ALS-C9Pos populations compared to cohorts of non-expanded ALS patients. Interestingly, the lower age at first observation and diagnosis described in our cohort of ALS-C9Pos patients was likely due to the higher attention to appearance of ALS symptoms revealed in subjects with known family history of ALS, as previously described in ALS-C9Pos patients (Umoh et al., 2016; Turner et al., 2017). Additionally, we reported an increased odds of bulbar onset in ALS-C9Pos patients, as also described in other cohorts of expanded patients (Chiò et al., 2012; Irwin et al., 2013; Cooper-Knock et al., 2014). This clinical onset has been used to identify a more aggressive ALS phenotype, since patients with bulbar disease may carry a worse prognosis (Magnus et al., 2002; Chiò et al., 2011) and peculiar neuropsychological and neuroimaging profiles (Cistaro et al., 2012; Trojsi et al., 2017a), as also confirmed by our results in which bulbar onset arises as an independent feature associated to *C9orf72* expansion and negatively impacting on prognostic outcome.

As expected, we revealed that ALS-C9Pos patients were more likely to exhibit FTD diagnosis and to report family history of ALS, FTD, and Alzheimer's disease, thereby confirming the previously identified clinical profile of ALS-C9Pos patients (Byrne et al., 2012, 2013; Chiò et al., 2012; Cooper-Knock et al., 2012, 2014; Sabatelli et al., 2012; Umoh et al., 2016; Hardiman et al., 2017). However, we established diagnosis of dementia in patients' relatives retrospectively from medical records or from patients' reports and this may have underestimated significant cognitive changes in their pedigrees. Moreover, in the studied ALS population the formal neuropsychological testing was performed using heterogeneous protocols among the different referral centers, without collecting cognitive and behavioral scores for each patient (Trojsi et al., 2017a).

Of note is also that in our population the *C9orf72* expansion was associated with lower odds of spinal onset, NIV, hypertension, and psychiatric diseases. This evidence was not unexpected with regard to the lower odds of association between the *C9orf72* expansion and spinal onset and NIV, in consideration of the above discussed higher odds of association between the *C9orf72* expansion and bulbar onset, among possible onsets, and given the higher association between the *C9orf72* expansion and dementia, proven to induce less adherence and compliance to treatments, including NIV (Govaarts et al., 2016; Mandrioli et al., 2018b).

The association between arterial hypertension and ALS has been previously investigated, resulting in conflicting evidence regarding the prognostic role of this comorbidity (Moreau et al., 2012; Körner et al., 2013; Moglia et al., 2017; Mandrioli et al., 2018a). In particular, some studies on large ALS clinic-based cohorts reported conflicting results on the association between premorbid or comorbid arterial hypertension and shorter survival in ALS patients (Körner et al., 2013; Mandrioli et al., 2018a). Probably, our result of a lower odds of association between *C9orf72* expansion and hypertension may be due to the lack of this comorbidity among clinical features potentially related to the *C9orf72*-mediated pathology, although hypertension was proven associated to shorter survival time in ALS-C9Pos patients on univariate analysis. To note, among the potential prognostic factors for survival in ALS, although we have no information regarding comorbid familial hypercholesterolemia (FH), 3.14% of ALSwoGM patients and 3.57% of ALS-C9Pos patients had hypercholesterolemia. In the light of the recent polygenic evidence that low-density lipoprotein cholesterol (LDL-C) and total cholesterol (TC) are causally associated with ALS (Chen et al., 2018), large-scale genome-wide association studies (GWASs) and deep sequencing for rare variants of LDL-C and TC risk alleles will be needed.

Additionally, the lower odds of association between the *C9orf72* expansion and psychiatric diseases found in our population, although apparently unexpected, may be explained on the basis of previous literature referring that behavioral abnormalities, rather than psychosis or other psychiatric syndromes *per se*, within clinical profile of FTD, were frequently observed among *C9orf72* expansion carriers (Watson et al., 2016; Devenney et al., 2017; Ducharme et al., 2017). Furthermore, as to the prevalence of neuropsychiatric conditions in relatives of ALS patients, although psychiatric symptoms have been described more frequently in ALS kindreds than in non-ALS pedigrees, the presence of *C9orf72* expansion was demonstrated not to fully account for this association (Byrne et al., 2013), suggesting that only some subphenotypes of ALS may share pleiotropic genetic risk with neuropsychiatric illnesses (O'Brien et al., 2017). Finally, there is some evidence that intermediate *C9orf72* repeat lengths are associated with personal or family history of FTD and/or psychiatric illness, although the “critical” *C9orf72* repeat size required for initiation of neurodegeneration remains unknown (Ng and Tan, 2017).

Our findings regarding the reduced overall survival in the ALS-C9Pos population compared to ALSwoGM patients and the significant associations between some clinical variables and shorter survival time in ALS-C9Pos patients are consistent, respectively, with previous evidence from other ALS-C9Pos cohorts compared to non-expanded ALS cohorts (Byrne et al., 2012; Chiò et al., 2012; Cooper-Knock et al., 2012; Irwin et al., 2013; Umoh et al., 2016; Hardiman et al., 2017; Rooney et al., 2017; Trojsi et al., 2017b) and from sporadic patients (Chiò et al., 2009; Watanabe et al., 2015). Interestingly, the multivariate analysis showed that shorter survival time was associated with the presence of *C9orf72* expansion in men when stratifying our ALS population by sex, thereby pointing toward the hypothesis that the male sex may drive the effect of the *C9orf72* repeat expansion rather than other prognostic factors, such as dementia and the clinical phenotype *per se*. To note, considering that the male sex could be more prone to certain clinical pathologies that decrease survival than the female one, to explore the differences in survival time between men and women, we performed a preliminary Kaplan-Meier analysis that did not show significant differences in the survival time between men and women for the overall sample, regardless of the stratification due to the presence of *C9orf72* repeat expansion (Supplemental Figure 1).

The result of our multivariate analysis, showing a shorter survival time in men carrying the *C9orf72* expansion, resembles what recently revealed in a combined analysis of the prognostic characteristics of the *C9orf72* repeat expansion, performed in 4925 ALS cases from Dutch, Irish, and Italian population-based national registers and from two (Belgian and UK) clinical research center cohorts (Rooney et al., 2017). In this study, Rooney et al. revealed a previously unrecognized interaction between the expanded variant and male patients with spinal onset disease who exhibited a shorter survival (Rooney et al., 2017). In comparison to our results on survival, the findings by Rooney et al. (2017) were derived from a more powered analysis, adequately sound to highlight interactions between the presence of *C9orf72* expansion and more demographic features, such as sex and site of onset. However, male sex emerges from both studies as a crucial interacting factor in the biology of *C9orf72*-mediated disease, in contrast to previous analyses performed in non-expanded ALS cohorts, that reported that female sex was an independent predictor of faster functional decline (Chiò et al., 2012; Watanabe et al., 2015). Nevertheless, the pathobiology of the observed interactions between both *C9orf72*- positive and -negative variants and sex remains still unclear. In this regard, the role of environmental risk factors should be emphasized, as underlined by a recent long-term population-based analysis from the Piemonte and Valle d'Aosta Register for ALS (PARALS), that showed that incidence of ALS increased in the last two decades, mostly in women. A probable explanation for this was derived from a birth cohort effect in women, who profoundly modified their lifestyle mainly from 1920, thereby being more exposed to possible environmental risk factors for ALS (i.e., physical activity and cigarette smoking) (Chiò et al., 2017). These results all together pointed to the potential pathogenic role of exogenous factors with different gender-effect, probably derived from interaction with specific genetic backgrounds. In case of *C9orf72* repeat expansion, several pathogenic mechanisms have been described, such as haploinsufficiency, toxic RNA interfering with the function of RNA-binding proteins or other cellular factors, presence of toxic dipeptide repeat proteins, and alterations of nucleocytoplasmic transport (Freibaum and Taylor, 2017). However, the potential interactions between these mechanisms and environmental risk factors for ALS have not been elucidated. Finally, the *C9orf72* repeat expansion is known to have an incomplete and age-dependent penetrance and, in this regard, a recent penetrance model analysis of a large cohort, drawn from the published literature, reported an older age of onset among female carriers in general, and among females with bulbar onset in particular (Murphy et al., 2017), thereby inducing to hypothesize potential hormonal or X-linked factors influencing disease type and site of onset.

Limitations of our study were the retrospective nature of the analysis, causing the unavailability of some data (such as the neuropsychological scores collected, the specific psychiatric manifestations, and the *C9orf72* repeat size) and the multicenter design of the study. Moreover, the sample size was relatively small, thereby implying that our study was not sufficiently powered to use regression models to explore the effects of known important survival covariates, including age of onset, site of onset, diagnostic delay, and *C9orf72*, and whether the expanded variant differentially affects outcome in ALS subgroups. In addition, the genetic panel shared by all participating centers was initially limited to screening *SOD1, C9orf72, TARDBP*, and *FUS* mutations, and was extended to other ALS- or FTD-related mutations only in familial cases. Specifically, in the absence of a family history of ALS, a case-control design, in which patient samples are compared with samples from people without ALS, has been recognized as the simplest approach for gene discovery, although requiring the ability to sequence the whole genome to identify the rare variation. As low-frequency variation seems to have a large role in the genetic architecture of ALS, including apparently sporadic ALS, looking for such variants has been shown crucial (Al-Chalabi et al., 2017). Increasing efforts have been made and will be further needed to understand the genetic component of ALS risk, especially carrying out large-scale international collaborations for exome analysis or more detailed genotyping of apparently sporadic patients.

Overall, we underlined that patients with ALS carrying the *C9orf72* repeat expansion exhibit remarkable clinical and pathological features suggesting that this hexanucleotide expansion identifies a distinct population of patients. In particular, the interaction between the *C9orf72* repeat expansion and gender should be further investigated to identify future disease-related prognostic models with potential, significant implications for screening of patients, and incorporation into clinical trials.

## Data Availability

The raw data supporting the conclusions of this manuscript will be made available by the authors, without undue reservation, to any qualified researcher.

## Ethics Statement

This study was approved by the Ethical Committees of the participating ALS centers and conducted according to the principles expressed in the Declaration of Helsinki. Written informed consent was obtained from all the participants of this study.

## Author Contributions

FT, MSi, CiF, GaS, CL, AnC, CM, KM, NT, ChF, CS, GiS, AmC, YF, RT, MR, VAS, AdC, GM, ViS, PV, CC, GQ, MSa, NR, GL, SM, AF, MM, GT, and JM conceived and designed the experiments. FT, MSi, CiF, GaS, CL, AnC, CM, KM, NT, ChF, CS, GiS, AmC, YF, RT, MR, VAS, AdC, GM, ViS, PV, CC, GQ, MSa, NR, GL, SM, AF, MM, and JM performed the experiments. FT, MSi, and GaS analyzed the data. FT, CiF, GaS, CL, AnC, CM, KM, NT, ChF, CS, GiS, AmC, YF, RT, MR, VAS, AdC, GM, ViS, PV, CC, GQ, MSa, NR, GL, SM, AF, MM, GT, and JM contributed reagents, materials and analysis tools. FT, MSi, GaS, CL, AnC, CM, KM, NT, ChF, CS, GiS, AmC, YF, RT, MR, VAS, AdC, GM, ViS, PV, GT, and JM wrote and/or revised the paper.

### Conflict of Interest Statement

The authors declare that the research was conducted in the absence of any commercial or financial relationships that could be construed as a potential conflict of interest.

## Supplementary Material

Supplemental Figure 1 Kaplan-Meier plots of survival probabilities, stratifying the whole sample by sex: no difference is displayed for cumulative survival in males [green line; median survival of 43 months (95% CI 39–47)] compared to females [blue line; median survival of 41 months (95% CI 37–45)]. Log-rank χ^2^ = 2.11, *p* = 0.149; +: censored cases.
