# Supplementary figures and images for: Comparative Analysis of C9orf72 and Sporadic Disease in a Large Multicenter ALS Population: The Effect of Male Sex on Survival of C9orf72 Positive Patients
Source: Front Neurosci. 2019 May 17;13:485. doi: 10.3389/fnins.2019.00485 (PMC6534038; doi:10.3389/fnins.2019.00485)

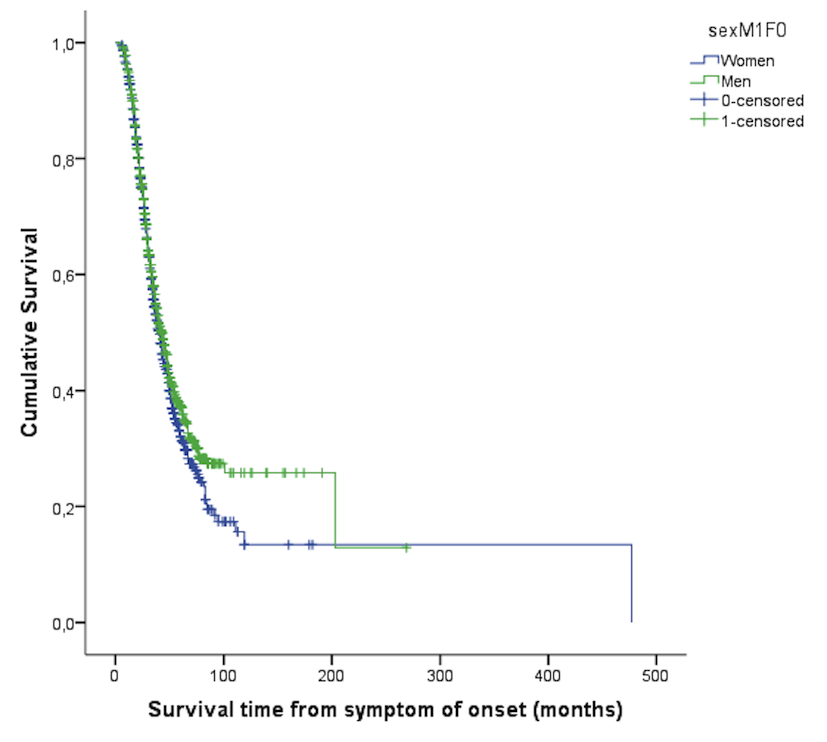

Supplement: Supplemental Figure 1 — Kaplan-Meier plots of survival probabilities, stratifying the whole sample by sex: no difference is displayed for cumulative survival in males [green line; median survival of 43 months (95% CI 39–47)] compared to females [blue line; median survival of 41 months (95% CI 37–45)]. Log-rank χ2 = 2.11, p = 0.149; +: censored cases. [file Image_1.TIFF]
