# Supplementary material for: Comparative Analysis of C9orf72 and Sporadic Disease in a Large Multicenter ALS Population: The Effect of Male Sex on Survival of C9orf72 Positive Patients
Source: Front Neurosci. 2019 May 17;13:485. doi: 10.3389/fnins.2019.00485 (PMC6534038; doi:10.3389/fnins.2019.00485)
Supplement: Supplementary file 2 [file Table_1.DOC]

**Supplemental Table 1.** Variables that were not included in the equation for each step of the Cox proportional hazards regression multivariate analysis (Forward Conditional method), performed in the whole sample, in men and women.

| **Variable** | **Score** | ***df*** | ***p*-value** |
| --- | --- | --- | --- |
| ***Whole sample*** |  |  |  |
| *Step 1* |  |  |  |
| Age at diagnosis | 129.05 | 1 | <.001 |
| Diagnostic delay | 129.05 | 1 | <.001 |
| Age at clinical observation | 60.21 | 1 | <.001 |
| Site of onset (Bulbar= 0; Spinal= 1) | 24.19 | 1 | <.001 |
| FTLD | 13.31 | 1 | <.001 |
| Hypertension | .00 | 1 | .972 |
| Psychiatric diseases | 3.23 | 1 | .072 |
| Family history of ALS | 11.31 | 1 | .001 |
| C9orf72 expansion status (No= 0; SI= 1) | 10.92 | 1 | .001 |
| *Step 2* |  |  |  |
| Age at diagnosis |  | 0 |  |
| Age at clinical observation | .18 | 1 | .670 |
| Site of onset (Bulbar= 0; Spinal= 1) | 12.02 | 1 | .001 |
| FTLD | 14.97 | 1 | <.001 |
| Hypertension | .25 | 1 | .616 |
| Psychiatric diseases | 1.38 | 1 | .239 |
| Family history of ALS | 7.93 | 1 | .005 |
| C9orf72 expansion status (No= 0; SI= 1) | 5.95 | 1 | .015 |
| *Step 3* |  |  |  |
| Age at diagnosis |  | 0 |  |
| Age at clinical observation | .25 | 1 | .611 |
| Site of onset (Bulbar= 0; Spinal= 1) | 10.18 | 1 | .001 |
| Hypertension | .23 | 1 | .633 |
| Psychiatric diseases | .58 | 1 | .447 |
| Family history of ALS | 4.96 | 1 | .026 |
| C9orf72 expansion status (No= 0; SI= 1) | 2.68 | 1 | .101 |
| *Step 4* |  |  |  |
| Age at diagnosis |  | 0 |  |
| Age at clinical observation | .24 | 1 | .626 |
| Hypertension | .25 | 1 | .617 |
| Psychiatric diseases | .58 | 1 | .445 |
| Family history of ALS | 4.40 | 1 | .036 |
| C9orf72 expansion status (No= 0; SI= 1) | 1.91 | 1 | .167 |
| *Step 5* |  |  |  |
| Age at diagnosis |  | 0 |  |
| Age at clinical observation | .27 | 1 | .60 |
| Hypertension | .20 | 1 | .65 |
| Psychiatric diseases | .85 | 1 | .35 |
| C9orf72 expansion status (No= 0; SI= 1) | .41 | 1 | .52 |
|  |  |  |  |
| ***Male*** |  |  |  |
| *Step 1* |  |  |  |
| Age at diagnosis | 57.06 | 1 | <.001 |
| Age at onset | 57.06 | 1 | <.001 |
| Age at clinical observation | 57.12 | 1 | <.001 |
| Site of onset (Bulbar= 0; Spinal= 1) | 10.74 | 1 | .001 |
| FTLD | 5.61 | 1 | .018 |
| Hypertension | 2.48 | 1 | .115 |
| Psychiatric diseases | .03 | 1 | .858 |
| Family history of ALS | 3.29 | 1 | .069 |
| C9orf72 expansion status (No= 0; SI= 1) | 3.13 | 1 | .077 |
| *Step 2* |  |  |  |
| Age at diagnosis | .25 | 1 | .620 |
| Age at onset | .25 | 1 | .620 |
| Site of onset (Bulbar= 0; Spinal= 1) | 7.99 | 1 | .005 |
| FTLD | 4.84 | 1 | .028 |
| Hypertension | .15 | 1 | .697 |
| Psychiatric diseases | .00 | 1 | .935 |
| Family history of ALS | 5.08 | 1 | .024 |
| C9orf72 expansion status (No= 0; SI= 1) | 6.46 | 1 | .011 |
| *Step 3* |  |  |  |
| Age at diagnosis | .25 | 1 | .619 |
| Age at onset | .25 | 1 | .619 |
| FTLD | 3.98 | 1 | .046 |
| Hypertension | .09 | 1 | .760 |
| Psychiatric diseases | .00 | 1 | .932 |
| Family history of ALS | 4.30 | 1 | .038 |
| C9orf72 expansion status (No= 0; SI= 1) | 5.46 | 1 | .019 |
| *Step 4* |  |  |  |
| Age at diagnosis | .18 | 1 | .67 |
| Age at onset | .18 | 1 | .67 |
| FTLD | 2.41 | 1 | .12 |
| Hypertension | .02 | 1 | .89 |
| Psychiatric diseases | .00 | 1 | .95 |
| Family history of ALS | 1.66 | 1 | .20 |
|  |  |  |  |
| ***Female*** |  |  |  |
| *Step 1* |  |  |  |
| Age at diagnosis | 62.69 | 1 | <.001 |
| Diagnostic delay | 62.69 | 1 | <.001 |
| Age at onset | 32.92 | 1 | <.001 |
| Site of onset (Bulbar= 0; Spinal= 1) | 12.35 | 1 | <.001 |
| FTLD | 6.77 | 1 | .009 |
| Hypertension | 1.37 | 1 | .240 |
| Psychiatric diseases | 1.97 | 1 | .160 |
| Family history of ALS | 3.07 | 1 | .080 |
| C9orf72 expansion status (No= 0; SI= 1) | 2.52 | 1 | .112 |
| *Step 2* |  |  |  |
| Age at diagnosis |  | 0 |  |
| Age at onset | .02 | 1 | .881 |
| Site of onset (Bulbar= 0; Spinal= 1) | 4.21 | 1 | .040 |
| FTLD | 12.91 | 1 | <.001 |
| Hypertension | 1.63 | 1 | .201 |
| Psychiatric diseases | 2.66 | 1 | .103 |
| Family history of ALS | 3.33 | 1 | .068 |
| C9orf72 expansion status (No= 0; SI= 1) | .94 | 1 | .331 |
| *Step 3* |  |  |  |
| Age at diagnosis |  | 0 |  |
| Age at onset | .06 | 1 | .805 |
| Site of onset (Bulbar= 0; Spinal= 1) | 2.88 | 1 | .089 |
| Hypertension | 1.36 | 1 | .243 |
| Psychiatric diseases | 1.38 | 1 | .239 |
| Family history of ALS | 1.80 | 1 | .179 |
| C9orf72 expansion status (No= 0; SI= 1) | .034 | 1 | .854 |

Note. df, degree of freedom; FTLD, frontotemporal lobar degeneration.
